# Susac Syndrome: an uncommon cause of impaired vision

**DOI:** 10.1007/s10072-022-05865-8

**Published:** 2022-01-10

**Authors:** Paolo Barbero, Domizia Vecchio, Eleonora Virgilio, Paola Naldi, Cristoforo Comi, Roberto Cantello

**Affiliations:** 1 Neurology Unit, Maggiore Della Carità Hospital, Department of Translational Medicine, University of Piemonte Orientale, Ospedale Maggiore Della Carità, via Mazzini 18, Novara, Italy; 2 Interdisciplinary Research Center of Autoimmune Diseases (IRCAD), Department of Health Sciences, University of Piemonte Orientale, Novara, Italy; 3 Neurology Unit, S. Andrea Hospital, Department of Translational Medicine, University of Piemonte Orientale, Vercelli, Italy

**Keywords:** Susac's syndrome, Encephalopathy, Hearing loss, Retinal occlusions

## Abstract

A 35-year-old Caucasian woman presented an abrupt onset of bilateral impaired vision, and arrived to our attention two weeks later. She had a previous episode of mild dizziness. She underwent a fluorescein angiography showing branch retinal artery occlusions and a brain magnetic resonance imaging (MRI) revealing several supraand infratentorial FLAIR-hyperintense white matter lesions, two with contrast enhancement. Thrombophilic, autoimmune and infective (including Human Immunodeficiency Virus, Borrelia burgdorferi, Hepatitis B Virus, Hepatitis C Virus, Herpes Simplex Virus 1-2, Varicella Zoster Virus) screening was negative. Cerebrospinal fluid analysis showed intrathecal IgG synthesis. We suspected a Primary Central Nervous System Vasculitis, and intravenous steroids were started. Three months later a second brain MRI showed seven new lesions without contrast enhancement, and she revealed a cognitive impairment and bilateral hearing loss. Reviewing the clinical history and MRI, she fulfilled diagnostic criteria for Susac syndrome. She had two cycles of cyclophosphamide, and recovered in 6 months and then remained stable with metotrexate.

## Case report

We present a 35-year-old woman who was admitted to our Neurological Department complaining bilateral impaired vision. Initially, visual symptoms started suddenly in the right eye and then progressed involving the left eye after four weeks. Her past medical history included a miscarriage and an extra-uterine pregnancy treated with salpingectomy after two regular pregnancies. Familiar history was negative, and she was on no medications. When asked, she revealed an episode of subjective vertigo lasting about one day, associated with nausea and vomiting, five weeks before. At the time, the patient was evaluated by her primary care physician and therefore treated with betahistine as a peripheral disorder with benefit.

Her first neurological examination showed impaired vision in both eyes assessed with Monoyer optometric chart (8/10 in the right eye and 6/10 in the left eye) with normal direct and consensual pupillary reflex. Muscle strength was globally normal, her deep tendon reflexes were brisk, and plantar responses bilaterally in flexion. Sensorial, cerebellar, higher cortical functions were normal.

**Fig. 1:**
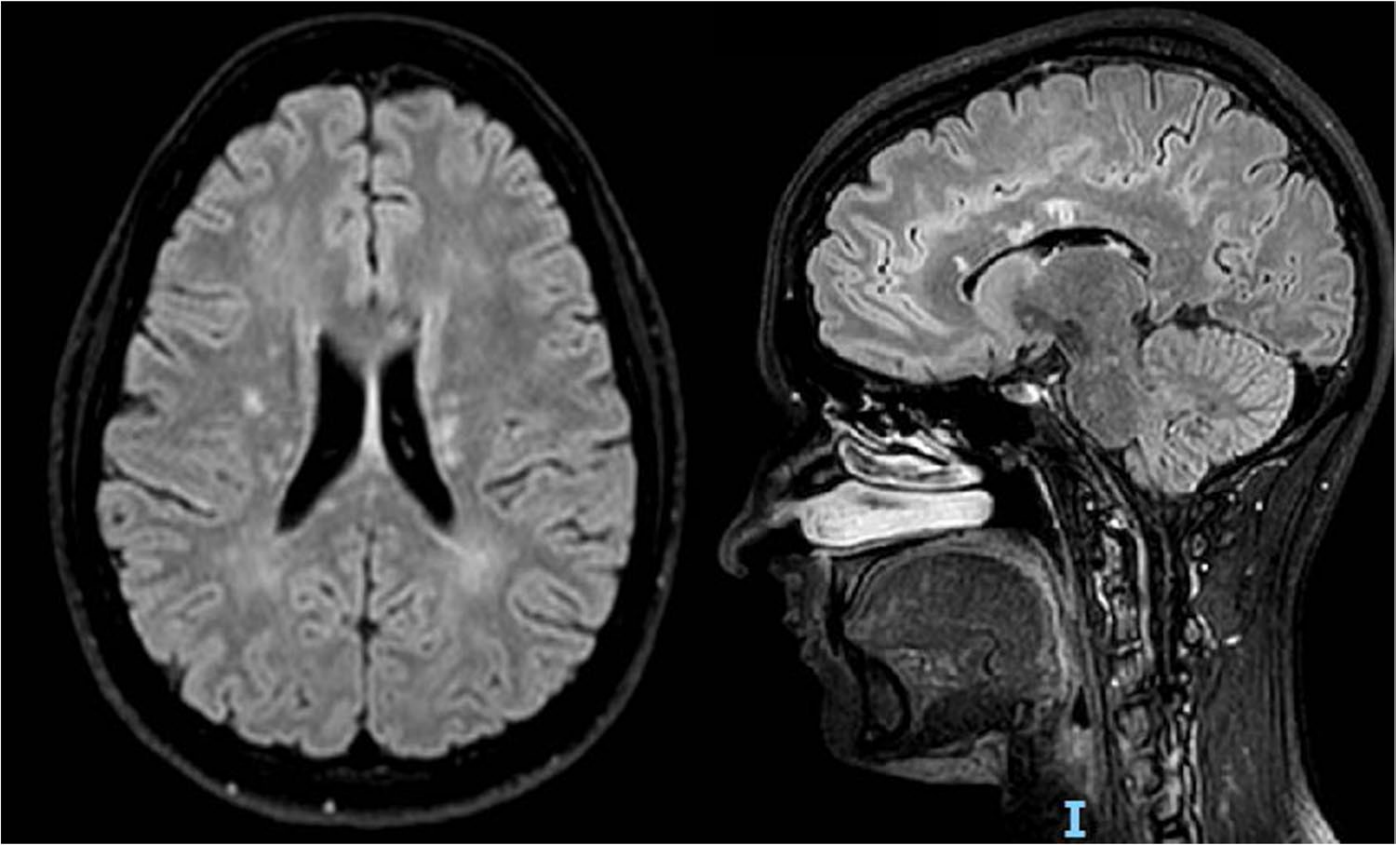
Brain MRI showing FLAIR hyperintense white matter lesions with "snowball"-like lesions in the corpus callosum

Before the admission, she had a fundoscopic examination followed by fluorescein angiography, showing ischemic retina with signs of branch retinal artery occlusion (BRAO) in right lower temporal artery, and was treated with acetylsalicylic acid 100 mg. A brain and spinal magnetic resonance imaging (MRI) revealed several FLAIR-hyperintense lesions, predominantly cortical and subcortical, and in white matter of left corona radiata, cerebellum, pons and middle cerebellar peduncles, two with contrast enhancement, and ten located to the corpus callosum (Fig. 1). The spinal cord was spared. She was largely investigated for vascular disorders with unremarkable results with: echocardiography, transcranial color Doppler, electrocardiogram, thrombophilic and autoimmune screening, including erythrocyte sedimentation rate, anti-neutrophil cytoplasmic, antinuclear, anti-double-stranded DNA and Lupus AntiCoagulant antibodies, proteins S, C and antithrombin III levels. An infective screening was negative including Human Immunodeficiency Virus, Borrelia burgdorferi, Hepatitis B Virus, Hepatitis C Virus, Herpes Simplex Virus 1–2, Varicella Zoster Virus, LUE and Quantiferon. A first lumbar puncture showed 9 lymphocytes/microL, mild protein elevation (119 mg/dL), kappa free light chain index of 20,5 and presence of oligoclonal bands. In the suspicion of a Primary Central Nervous System (CNS) Vasculitis, we started methylprednisolone 1000 mg for five days followed by a slow tapering to prednisone 75 mg/die, associated with intravenous immunoglobulins (0,4 g/kg/die for five days). Acetylsalicylic acid was switched to clopidogrel 75 mg. The patient did not recover significantly at discharge.

**Fig. 2:**
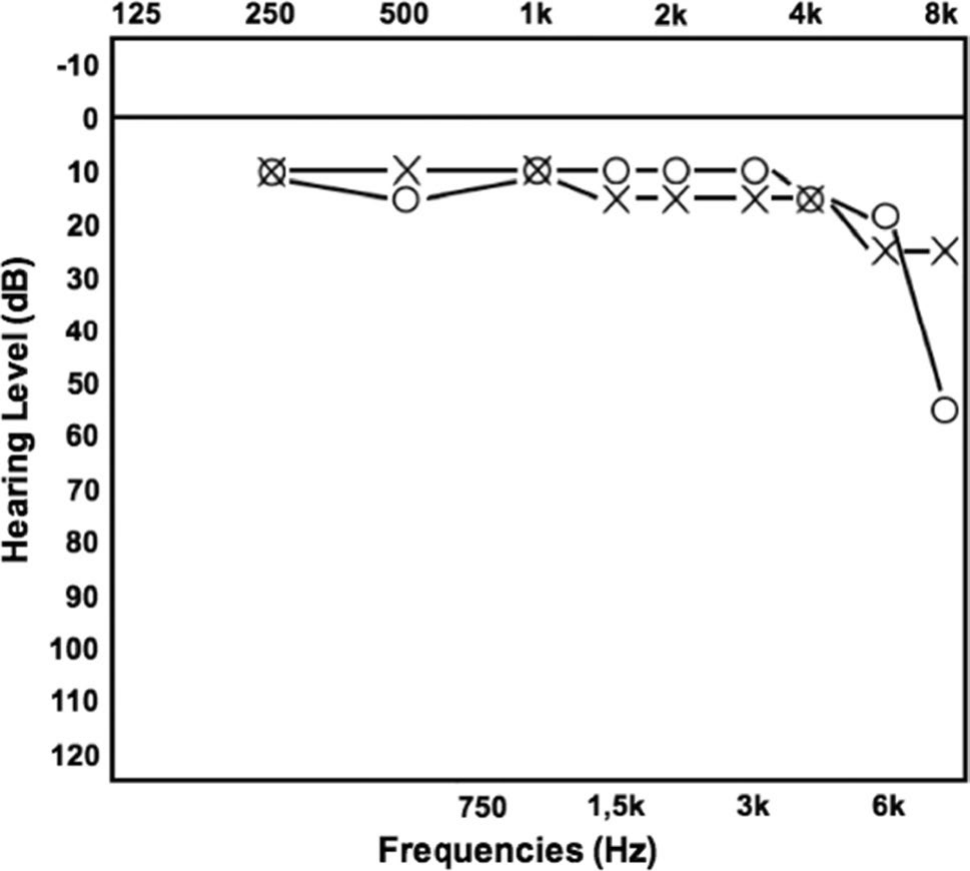
Audiometry evaluation revealing a right loss of the middle frequencies

Three months later, she presented a bilateral hearing loss, and a cognitive impairment confirmed by the Montreal Cognitive Assessment (total score 23/30) and Symbol Digit Modalities Test (t-score 33) that showed short-time memory lost, attention and verbal fluency deficit. Audiometry evaluation revealed a right loss of the middle frequencies (Fig. 2). A brain MRI showed seven new bilateral white matter lesions in right corona radiata, left frontal white matter, internal capsule and lenticular nucleus, without contrast enhancement. We repeated a lumbar puncture that confirmed mild protein elevation (70,1 mg/dL). At that time, we suspected Susac syndrome and, reviewing the clinical history and MRI, the patient fulfilled the diagnostic criteria of European Susac Consortium (EuSaC): 1) headache and cognitive impairment with hyperintense, multifocal, round small lesions involving also the corpus callosum, 2) vision loss with BRAO and 3) bilateral middle-frequency hearing loss at the audiogram [1].

According to the clinical and radiological worsening on steroids, cyclophosphamide 1 g/m^2^/month was started, with an improvement of the cognitive impairment and partial resolution of the brain lesions located in pons, internal capsule and basal ganglia three months later. She repeated after six months a second course of cyclophosphamide, being on steroids 60 mg daily meantime. We then tapered till stopping the steroids after introducing a maintenance immunosuppressant therapy: first azathioprine 50 mg, interrupted after three weeks due to gastrointestinal adverse effects and high lipase levels, then metotrexate 15 mg/week. She continued also clopidogrel. Over the next months, she clinically recovered except for a bilateral minor visual loss (9/10 in both eyes). Fluorescein angiography was planned but not performed (the patient had malaise and stopped the examination), and she described a recovery in the auditory function. She returned to her personal and working life as a nurse without clinical and radiological relapses over the next two years.

## Discussion

Susac syndrome (SuS) is a rare immune-mediated endotheliopathy that affects the small arterioles of the brain, retina and inner ear. The involvement of the three systems characterizes the “complete” triad of encephalopathy, BRAO and sensori-neural hearing loss [2] that could develop over years. Subclinical pathology occurs in a substantial number of patients sometimes causing a delay in diagnosis. In many patients, headaches (including migraine-like) precede the development of other symptoms and additional neurological findings, such as: cognitive dysfunction, gait disturbance and slurred speech [3]. As concerns the visual deficit, the patient presents a prompt “dark spot” or “black area” in the visual field that could permanently impair vision. The field defects are altitudinal and uncommonly involve the blind spot [4]. Finally, an asymmetrical and relatively sudden hearing loss for low and mid-frequencies could evolve to severe hypoacusia (because of a damage to cochlea) and be associated to dizziness (in relation to the vestibular apparatus) [5].

SuS incomplete presentation [6, 7] could resemble multiple sclerosis, cerebrovascular disease and primary Central Nervous System vasculitis, connective tissue disease or infections [8]; therefore, blood and CSF analysis are useful to exclude other differentials. Brain MRI, fluorescein angiography and audiometry are essential for correct diagnosis [8]. Typical MRI findings include multiple hyper-intense small lesions through infratentorial structures with involvement of the corpus callosum. Nonetheless, callosal lesions, called “snowball lesions", typically involve the central fibers [9] and are useful to differentiate from multiple sclerosis. In our case the development of “complete” triad of encephalopathy, BRAO and sensori-neural hearing loss suggested SuS.

To our knowledge, there are no trials on SuS [10] although these cases must be treated early, aggressively, and for a sufficiently long time to prevent relapses [11].

According to Fox et al. [12], we treated immediately with steroids, starting methylprednisolone (1000 mg/day for 5 days), in association with intravenous immunoglobulins (2 g/kg in 4 days). Moreover, as suggested, we added an antiplatelet therapy.

**Fig. 3:**
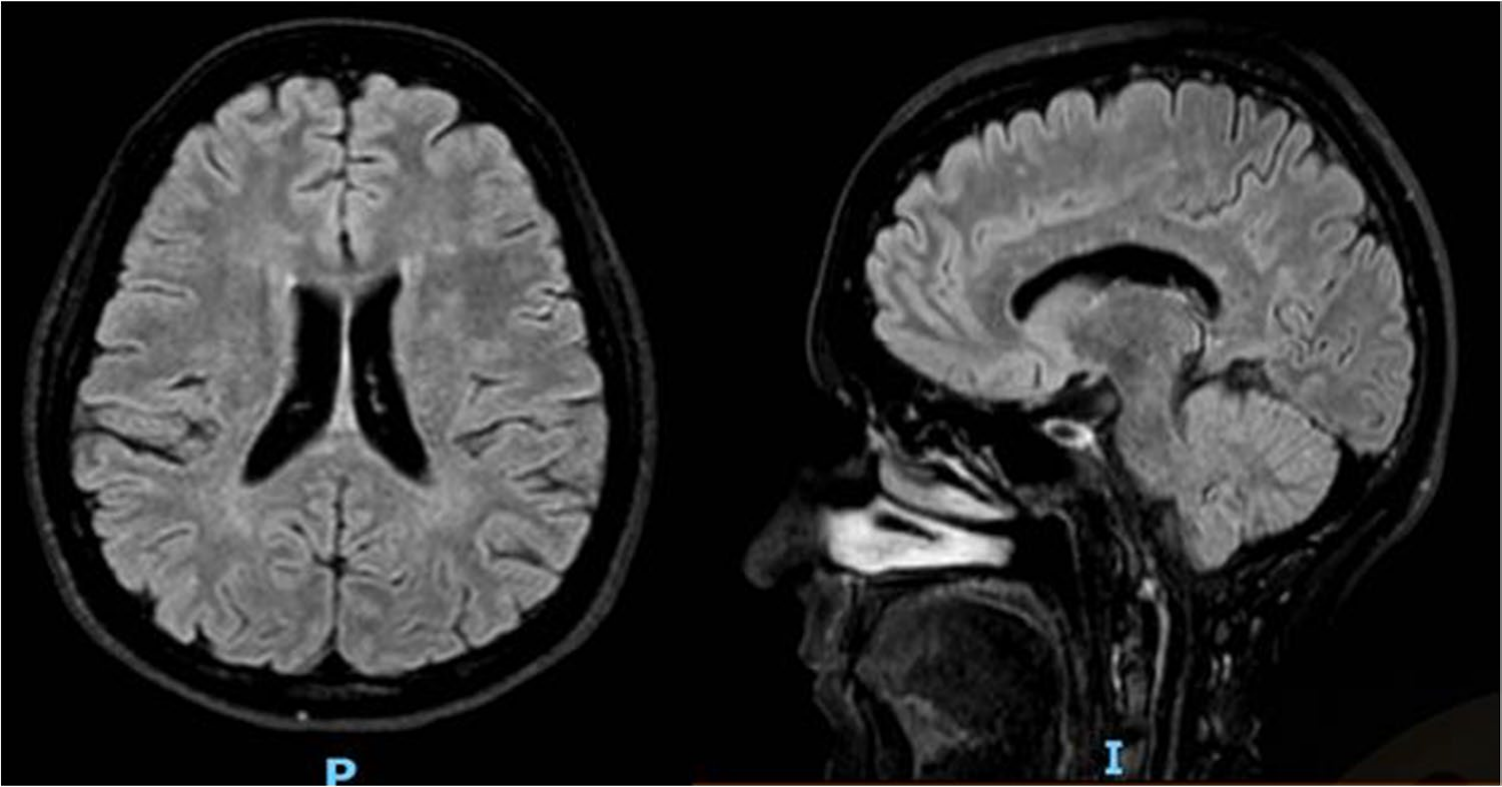
Last brain MRI, showing improvement of radiological findings

There is no clear indication in starting immunosuppressive therapies, except severe and debilitating cases, for whom cyclophosphamide, mycophenolatmofetil or rituximab should be added to glucocorticosteroids [13]. In our case, because of the cognitive and radiological worsening, we started cyclophosphamide that led to a recovery, followed by maintenance therapy with metotrexate, considering the risk of BRAO recurrence [14]. The patient had a regular and prolonged follow-up that included only brain MRI (Fig. 3), neurological and rheumatologic evaluations.

## Conclusion

Only the 13% of SuS patients presents the complete clinical triad at disease onset, so clinicians must search for subclinical signs. A prompt immunosuppressive treatment may decrease the risk of permanent neuropsychological, visual or auditive deficits in young patients [15, 16].
